# Combination of lyophilized adipose-derived stem cell concentrated conditioned medium and polysaccharide hydrogel in the inhibition of hypertrophic scarring

**DOI:** 10.1186/s13287-020-02061-3

**Published:** 2021-01-07

**Authors:** Chaoyu Zhang, Ting Wang, Li Zhang, Penghong Chen, Shijie Tang, Aizhen Chen, Ming Li, Guohao Peng, Hangqi Gao, Haiyan Weng, Haoruo Zhang, Shirong Li, Jinghua Chen, Liangwan Chen, Xiaosong Chen

**Affiliations:** 1 Department of Plastic Surgery, Fujian Medical University Union Hospital, Fuzhou, China; 2 Department of Plastic Surgery and Regenerative Medicine Institute, Fujian Medical University, Fuzhou, China; 3 Department of Stem Cell Research Institute, Fujian Medical University, Fuzhou, China; 4 Department of Central Sterile Supply, Fujian Medical University Union Hospital, Fuzhou, China; 5 Department of Plastic and Reconstructive Surgery, Southwestern Hospital, Army Military Medical University, Chongqing, China; 6 Department of Pharmaceutical Analysis, The School of Pharmacy, Fujian Medical University, Fuzhou, China; 7 Department of Cardiac Surgery, Fujian Medical University Union Hospital, Fuzhou, China

**Keywords:** Adipose-derived stem cells, Conditioned medium, Freeze-drying, Hydrogel, Scar hyperplasia

## Abstract

**Background:**

Mesenchymal stem cell-based acellular therapies have been widely exploited in managing hypertrophic scars. However, low maintenance dose and transitory therapeutic effects during topical medication remain a thorny issue. Herein, this study aimed to optimize the curative effect of adipose-derived stem cell conditioned medium (ADSC-CM) in the prevention of hypertrophic scarring.

**Methods:**

In the present study, ADSC-CM was concentrated via the freeze-drying procedure. The efficacy of different dose groups (CM, CM5, CM10) was conducted on the proliferation, apoptosis, and α-smooth muscle actin (α-SMA) expression of human keloid fibroblasts (HKFs) in vitro. Incorporation of adipose-derived stem cell concentrated conditioned medium (ADSCC-CM) into polysaccharide hydrogel was investigated in rabbit ear, in vivo. Haematoxylin-eosin (H&E) and Masson’s trichrome staining were performed for the evaluation of scar hyperplasia.

**Results:**

We noted that ADSCC-CM could downregulate the α-SMA expression of HKFs in a dose-dependent manner. In the rabbit ear model, the scar hyperplasia in the medium-dose group (CM5) and high-dose group (CM10) was inhibited with reduced scar elevation index (SEI) under 4 months of observation. It is noteworthy that the union of CM5 and polysaccharide hydrogel (CM5+H) yielded the best preventive effect on scar hyperplasia. Briefly, melanin, height, vascularity, and pliability in the CM5+H group were better than those of the control group. Collagen was evenly distributed, and skin appendages could be regenerated.

**Conclusions:**

Altogether, ADSCC-CM can downregulate the expression of α-SMA due to its anti-fibrosis effect and promote the rearrangement of collagen fibres, which is integral to scar precaution. The in situ cross bonding of ADSCC-CM and polysaccharide hydrogel could remarkably enhance the therapeutic outcomes in inhibiting scar proliferation. Hence, the alliance of ADSCC-CM and hydrogel may become a potential alternative in hypertrophic scar prophylaxis.

**Supplementary Information:**

The online version contains supplementary material available at 10.1186/s13287-020-02061-3.

## Background

Hypertrophic scar (HS) is characterized by fibroblast over-growth and excessive secretion of the extracellular matrix [1], which is a typical prognosis of tissue regeneration following dermal injuries. The hypertrophic scarring response has a pathological spectrum, ranging from aesthetic perplexity to significant functional damage. To date, there is fairly general agreement that customized precaution is recommended for the hypertrophic scar formation regarding the inconclusive proof of clinical outcomes [2].

Due to the latest progress in stem cell research, acellular therapy based on stem cells provides a potential alternative strategy for excessive scar formation. Multiple studies have confirmed that adipose-derived stem cells (ADSCs) and their derivatives are highly applicable to scar hyperplasia prevention [3–5]. Mainly, adipose-derived stem cell conditioned medium (ADSC-CM) derives significant benefits from the advantages of easy access, convenient utility, and high security [6], which may seem an outperformer in stem cell therapy.

However, the scarcity of cytokines in stem cell-conditioned medium limits its efficacy. In this study, we describe the possibility of utilizing freeze-dried ADSC-CM as a practical option for the enrichment of the stem cell paracrine cytokines. Freeze-drying could be exploited as an avenue of long-term preservation of paracrine cytokines [7], which optimized the storage mode of conditional medium simultaneously.

Nevertheless, as nano-sized particles, functional proteins originated from stem cells could be deprived of their benefits due to low retention rates and could not be preserved solely in the wound surface for a continuous period [8]. To prolong the action time of cytokines on the wound surface, we formed a semi-solid drug reservoir through the alliance of adipose-derived stem cell concentrated conditioned medium (ADSCC-CM) and the polysaccharide hydrogel aiming to exert its sustained-release effect. Currently, polysaccharide hydrogel has emerged as a scaffold material with good biocompatibility while achieving widespread adoption into the clinical realm by virtue of its tunable morphology, controllable degradation, and release behaviour [9–11]. In situ gelling on wounds demonstrated considerable prospects in achieving an accurate fit with irregular shape tissue defects. Hydrogel allows moisture retention with minimized exudate leaks, which could play a potentially critical role in wound protection [12]. Moreover, the loose and porous structure in the gel contributes to the slow release of cytokines in the conditioned medium [13].

Herein, we proposed freeze-drying as a potential method to improve ADSC-CM potency, and the appropriate concentration in hypertrophic scarring alleviation was explored in vivo and in vitro. Furthermore, the polysaccharide hydrogel was combined, hoping to prolong the therapeutic effect of cytokines. The alliance of ADSCC-CM and hydrogel was studied in the hypertrophic scar prophylaxis using the rabbit ear model (Additional file 1: Figure S1).

## Materials and methods

### Animal maintenance

All animal protocols were implemented under the Animal Ethical Committee of Fujian Medical University’s supervision and approval (Permit Number: FJMU IACUC 2018-089). Twelve male New Zealand rabbits (3 months of age) were raised in the Experimental Animal Center of Fujian Medical University. Animals were kept in cages individually after wounding and maintained under ambient temperature.

### Acquisition of rabbit adipose-derived stem cells (rADSCs)

After euthanatized, the inguinal fat tissues of rabbits were collected. Then, specimens were shredded and digested with 0.125% type I collagenase (Biofroxx, Guangzhou, China) at 37 °C for 45 min. The pellet was filtered successively with 100 μm and 40 μm stainer and centrifuged with 400*g* for 5 min. Finally, the cell suspension was cultured in a 5% CO_2_ cell incubator at 37 °C with low-glucose Dulbecco’s modified Eagle’s medium (DMEM) (HyClone, UT, USA) containing 10% foetal bovine serum (FBS) (Gibco, CA, USA) and 1% penicillin/streptomycin (Sigma-Aldrich). The culture medium was changed every 3 days.

### Identification of rADSCs

The expression of cell surface markers was identified by the FCM method using CD11b (Abcam, Cambridge, UK), CD44 (Thermo, MA, USA), CD90 (BioLegend, CA, USA), and HLA-DR (BD, NJ, USA). BD FACS Celesta™ flow cytometer (BD, CA, USA) was used for detection. After induced by adipogenic and osteogenic induction solutions respectively for 21 days, the stem cell differentiation ability was detected by Oil Red Assay and Alkaline Phosphatase Assay (KeyGEN, Jiangsu, China).

### Concentration of ADSC-CM

The 4th passage of ADSCs was selected. For the T75 culture flask, 10 ml serum-free culture medium was added standardly. When the cell adhesion fusion rate reached 80%, serum-free starvation culture was initiated for 48 h [14]. The conditioned medium was collected and centrifuged at 1000*g*, 15 min for removing cell fragments, and a 0.22-μm filter was used to eliminate the existing bacteria. A vacuum freeze dryer (LC, Shanghai, China) was employed for the complete lyophilization of ADSC-CM for 12 h. The concentration of ADSC-CM was attained through rehydration of the freeze-dried powder with appropriate volumes of DMEM.

### Detection of total protein in ADSCC-CM by bicinchoninic acid (BCA) assay

The BCA protein detection kit (Beyotime, Shanghai, China) was used to detect the total protein of the ADSCC-CM, and DMEM were exploited in the control group. The experiment was carried out following the manufacturer’s guidance. The total protein of tested samples was calculated from a standard curve.

### Configuration of polysaccharide hydrogel

VitroGel 3D-RGD (the Well, NJ, USA) and 1× DPBS (HyClone, UT, USA) were mixed at a ratio of 1:1 for dilution. The diluted VitroGel 3D was then combined with ADSCC-CM to prepare semi-solid hydrogel in a syringe. As an ion-crosslinking hydrogel, the cross-bonding process was initiated by the mixture of culture medium and hydrogel [15]. The semi-solid gel was gradually formed at 4 °C for 10 min for further administration to the wound surface.

### Detection of human keloid fibroblasts (HKFs) proliferation by CCK-8

Cell suspensions were inoculated in 96-well plates with a density of 4000 cells per well. The growth of HKFs (CRL-1762™, ATCC) was calculated after intervention by different doses of conditioned medium for 0, 24, and 48 h. CCK-8 (Med, Shanghai, China) was applied to each well and incubated at 37 °C for 2 h. The Multiskan™ FC microplate reader was used to measure the absorbance of the samples.

### Detection of HKFs apoptosis by annexin V/PI double staining

After co-incubating with the conditioned medium for 48 h, apoptosis of HKFs was detected by annexin V/PI double staining (Beyotime, Shanghai, China). The operation was conducted based on the instructions. The BD Accuri C6 Plus Flow Cytometer (BD Biosciences, San Jose, CA, USA) was utilized for further analysis.

### Detection of α-smooth muscle actin (α-SMA) expression in HKFs

CytoFix/Cytoperm™ (BD, NJ, USA) was selected to fix and perforate the cells under incubation at 4 °C for 20 min. The pallet was stained with the primary antibody against α-SMA (1: 2000; Abcam, UK) for 30 min. The cells were resuspended with PBS, centrifuged at 300*g* for 5 min, and incubated with goat anti-mouse IgG-Alexa Fluor® 488 (1:1000; Abcam, UK) for 30 min. The fluorescence intensity was detected by the BD Accuri C6 Plus™ Flow Cytometer (BD, CA, USA). The DMI fluorescence microscope (Leica, Germany) was employed for fluorescence imaging.

### Establishment of the hypertrophic scar model

The in vivo experiments were divided into the gel group and the non-gel group. The gel group was treated with ADSCC-CM combined with polysaccharide hydrogel, and DMEM mixed with hydrogel was given in the control group. The non-gel group was applied with ADSCC-CM, and DMEM was used in the control group. After intraperitoneal injection of 2% pentobarbital sodium 40 mg/kg, the full-thickness skin and perichondrium were removed along the long axis of the ventral middle part of the rabbit ear to make a round defect with a diameter of 1 cm under aseptic operation. Four identical wound defects were made on each side of the rabbit ears removing all layers of skin and perichondrium [16]. Each rabbit was injected with gentamicin intramuscularly to prevent postoperative infection. Any wound with signs of infection or necrosis would be excluded from the study.

### SEI measurements

Scar elevation index (SEI) is an accurate and reproducible measurement for evaluation of hypertrophic scarring [17, 18], which can be referred to as the ratio of the total tissue thickness above the cartilage surface in the wound area to the normal tissue thickness above the cartilage surface. Among them, SEI = 1 indicates that the height of the scar is equal to that of the surrounding uninjured dermis, and SEI > 1 is representative of hypertrophic scar. Randomized, double-blind studies were carried out for SEI measurement of the histological specimens utilizing the ImageJ software (version 1.52a, Bethesda, MD, USA).

### Macroscopic observation of scar

Sodium chloride was chosen for wound cleaning. The gross view of the wound was photographed with Canon EOS 5D3 and EF 24–70 mm lens in multiple periods of 0, 2, 7, 14, 28, 56, and 84 days postoperative, and wound healing and scar hyperplasia were recorded. Due to the thin layer of rabbit ears, a vital light source was given from the reverse side to observe the angiogenesis around the wound straightforwardly.

### H&E and Masson staining

At 16 weeks after the operation, rabbits were sacrificed by CO_2_ asphyxiation, and scar tissues were cut into two halves from the highest point for histological examination. The specimens were utterly soaked and fixed with 4% paraformaldehyde (Solarbio, Beijing, China) for 48 h. After dehydration, the samples were embedded in paraffin, sliced, and stained with H&E. The accumulation and arrangement of collagen in scar tissue were observed by Masson’s trichrome staining. The DM2500 fluorescence microscope (Leica, Germany) was utilized for images taken with the magnification of 50–200 times.

### Protein mass spectrometry detection of ADSC-CM

Two samples of the 4th-generation human ADSC-CM were selected and quickly frozen in liquid nitrogen for 30 min and then transferred to − 80 °C for subsequent storage. Shotgun LC-MS/MS analysis [19] was carried out for the protein mass spectrometry detection. Sequentially, the application of the Mascot software (version 2.2) was manipulated for searching the corresponding database of mass spectrometry and identified proteins were matched via the Uniprot database [20]. Ultimately, the top 50 proteins with a high relative abundance (Σ#PSMs) were analysed in the field of tissue regeneration and scar hyperplasia.

### Statistical analysis

The quantitative data were expressed as mean ± standard deviation with *p* < 0.05 considered as a significant difference. GraphPad Prism (version 8.02, La Jolla, CA) was chosen for charting and statistical analysis. Normal distribution was identified through the analysis of the D’Agostino and Pearson omnibus normality test and Kolmogorov-Smirnov test. The Student *t* test was selected for comparison between the two groups. One-way ANOVA was used for multi-group comparison. Two-way ANONA was suitable for bivariate analysis.

## Results

### Properties of lyophilized ADSC-CM and injectable hydrogel

**Fig. 1:**
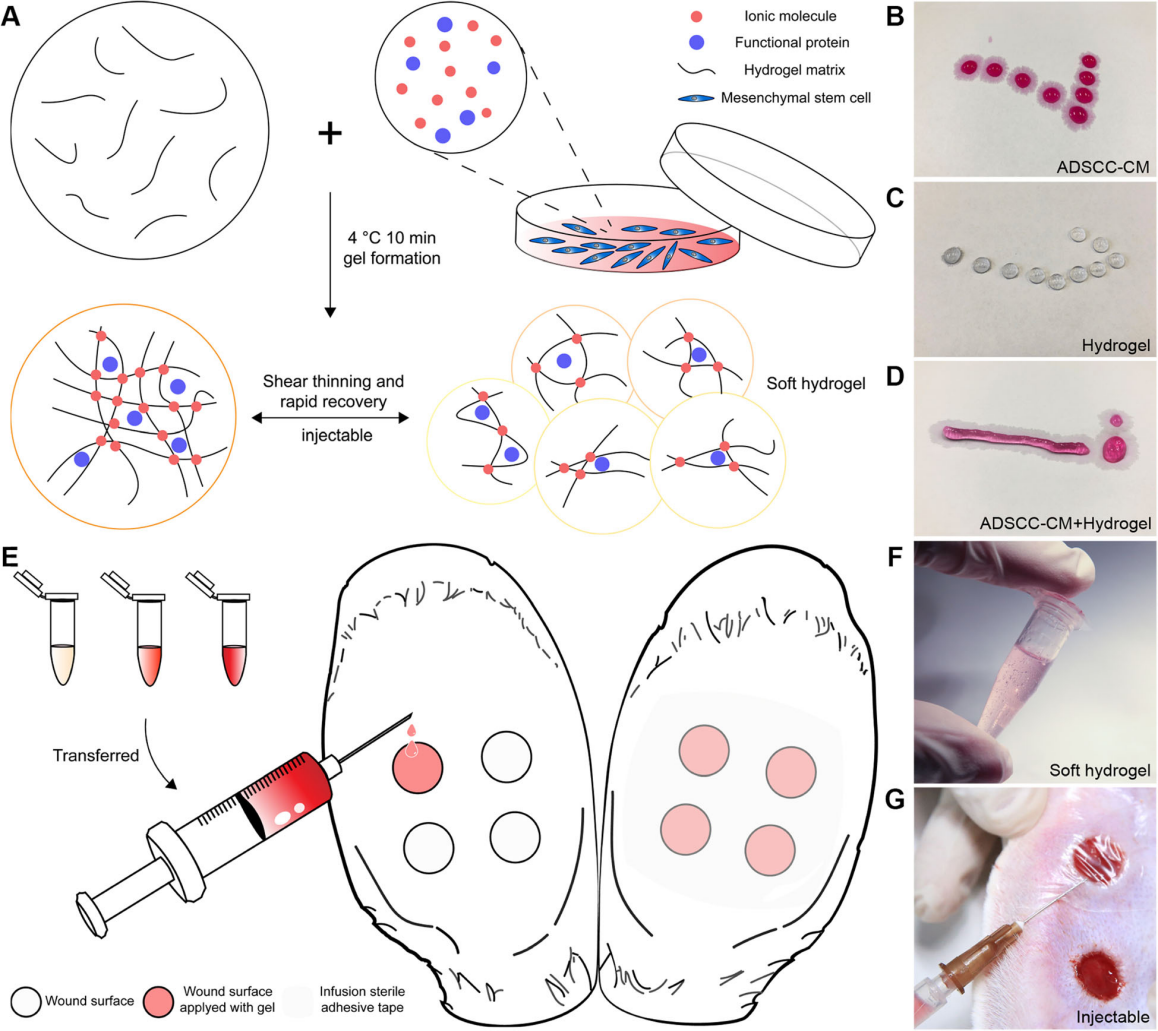
Preparation and intervention process of ADSCC-CM combined with hydrogel. **a** During injection, the hydrogel matrix was broken into small pieces forming a soft hydrogel. When the interference of shear force was eliminated, the structure of hydrogel could be recovered. **b**–**d** The gel was liquid before adding the cell culture medium. After mixing with the culture medium at 4 °C for 10 min, the gel could be semi-solid. **e**–**g** Four identical wounds of 1.0 × 1.0 cm were prepared for each ear with a punch. The soft hydrogels’ injectable property rendered it practisable to transfer to the injured site. And sterile infusion adhesive tapes were used to restrain ADSCC-CM from flowing out

After complete lyophilization, ADSC-CM would represent the form of a uniform fine powder, with basically identical weight (Additional file 1: Figure S2). By comparing the weight of the ADSC-CM before and after freeze-drying, we initially verified the stability of the freeze-drying process. The hydrogel is aqueous before adding the cell culture medium. The ionic molecules in the cell culture medium help to connect the short nanofibres end to end to elongate nanofibres which are further cross-linked into a reticular structure. Mixture with the ADSCC-CM at 4 °C for 10 min helps to increase the strength of the cross-linking of polysaccharide hydrogel, thus making a semi-solid drug reservoir of stem cell paracrine protein (Fig. 1a–d). Owing to the soft hydrogel’s injectable property and its slow gelatinization process, we were able to transfer the gel from the mixing tubes to the injured site (Fig. 1e–g).

### Characteristics of rADSCs

**Fig. 2:**
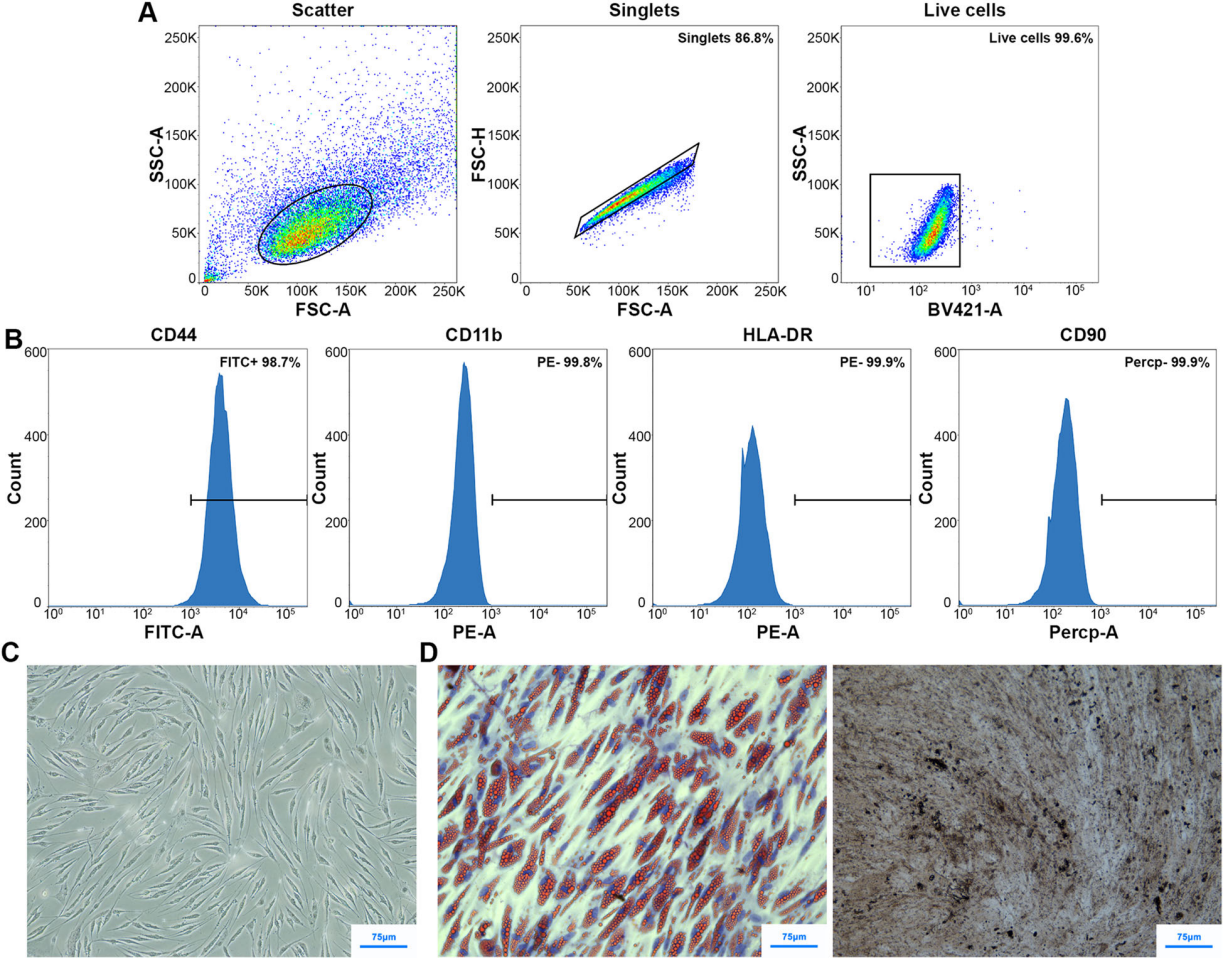
Characteristics of rADSCs. **a**, **b** Adherent cells (13.2%) and dead cells (0.4%) were excluded from the primary cell group. Flow cytometry analysis of the cell surface markers CD44, CD11b, HLA-DR, and CD90 was shown in rADSCs. **c** The morphology of rADSCs under a microscope. **d** Alkaline phosphatase and Oil Red O staining for differentiated rADSCs

The expression of surface markers of rADSCs at P4 was evaluated by flow cytometry. We performed a follow-up analysis on the premise that 99% of the cells in the sample were live cells, and no adhesion cell mass was contained. As a result, CD44 was highly expressed, while HLA-DR, CD90, and CD11b were negatively expressed (Fig. 2a, b). Under the light microscope, rADSCs demonstrated homogeneous morphology of fibroblast-like spindle-shaped (Fig. 2c). After adipogenic induction, the transparent lipid droplets in the cell could be stained red through Oil Red O staining. After osteogenic induction, the characteristic black cobalt sulfide precipitation could be observed through alkaline phosphatase staining (Fig. 2d). The results suggested that rADSCs have adipogenic and osteogenic differentiation capabilities.

### ADSCC-CM promotes the proliferation of HKFs and inhibits apoptosis

**Fig. 3:**
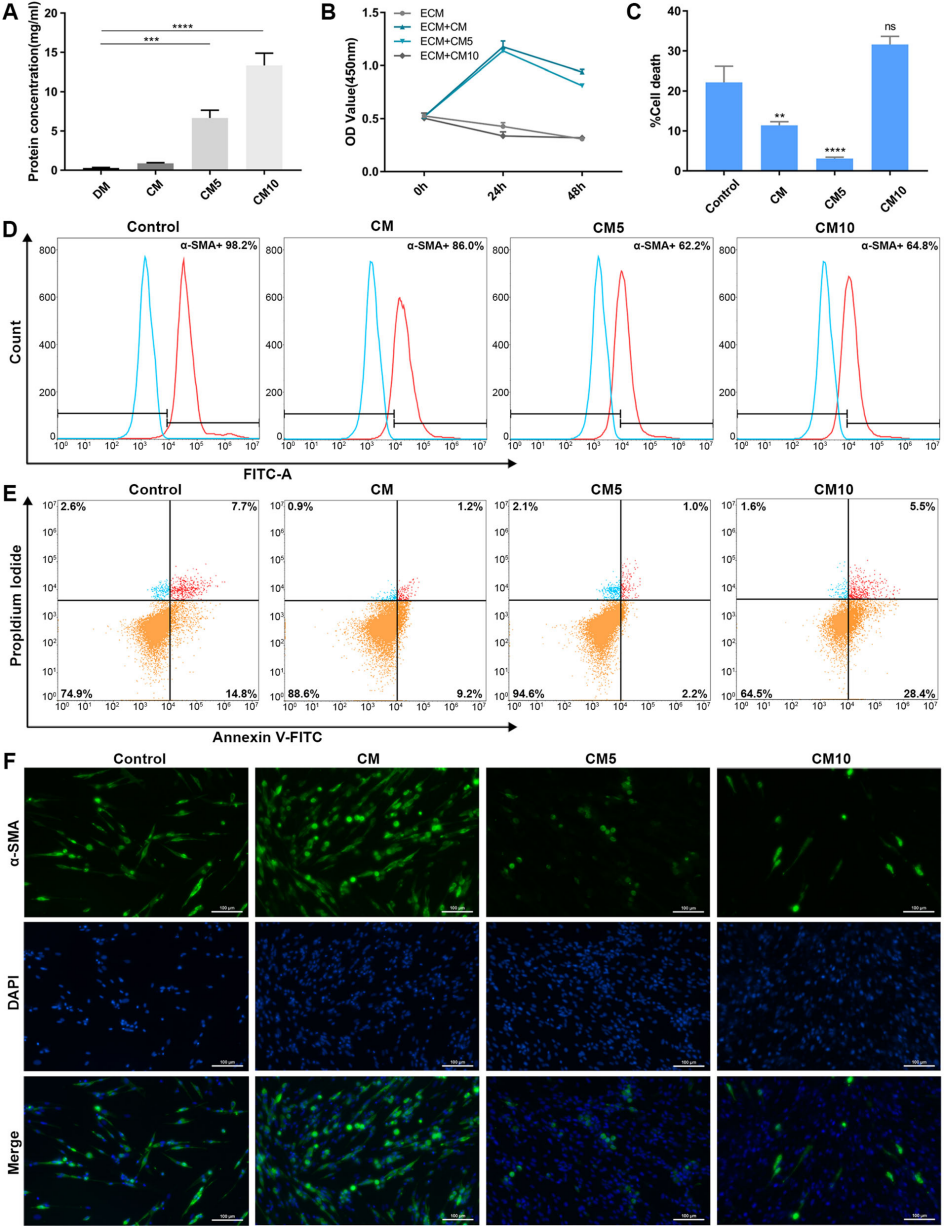
Effect of ADSCC-CM on HKFs. **a** The total protein detection of CM, CM5, and CM10. **b** The proliferation of HKFs was detected after CM, CM5, and CM10 intervention for 0 h, 24 h, and 48 h, respectively. DM was set as control. **c**, **d** HKFs were treated with CM, CM5, and CM10 for 48 h (*n* = 3). Apoptosis was detected. **e** HKFs were treated with CM, CM5, and CM10 for 48 h (*n* = 3). The expression of α-SMA in HKFs was detected by flow cytometry. **f** Immunocytochemical of fibroblasts: α-SMA was labelled with Alexa Fluor®488 (green), and the nucleus was stained with DAPI (blue). DAPI, 4′,6-diamidino-2-phenylindole

Overactivation of fibroblasts is considered the core of scar hyperplasia, which is closely related to their capacity to secrete collagen [21]. Since keloid fibroblasts are a kind of “activated” cells [22], we choose HKFs as the target cells of scarring in vitro studies. The BCA assay detected the total protein of ADSCC-CM. It could be inferred that the complete protein in the CM5 and CM10 was elevated considerably (Fig. 3a). We confirmed that the viability of HKFs in the CM group and CM5 group was remarkably increased compared with the control group. The growth of fibroblasts was hampered by further elevating the conditioned medium concentration (Fig. 3b). Apoptosis decreased in the CM and CM5 groups but increased in the CM10 group (Fig. 3c, d). Together, CM and CM5 could promote cell growth and reduce cell death within 48 h, while CM10 would inhibit HKF proliferation and activate apoptosis.

### ADSCC-CM inhibits the expression of α-SMA in HKFs

α-SMA related to the TGF-β/Smad2 signalling pathway activity participates in the transition of fibroblasts/myofibroblasts [23]. Our experiment elegantly indicated that over 95% proportion of HKFs were α-SMA positive. For further study, the fibroblasts were co-cultured with different concentrations of ADSCC-CM (CM, CM5, CM10) for 48 h. A visible descending of α-SMA was found in the CM5 and CM10 groups (Fig. 3e). To corroborate these in vitro findings, we use immunofluorescence to detect α-SMA of HKFs in situ, yielding a similar outcome (Fig. 3f). The alleviation of α-SMA expression illustrates a suppressive role in scarring.

### The effect of ADSCC-CM combined with polysaccharide hydrogel on wound healing

**Fig. 4:**
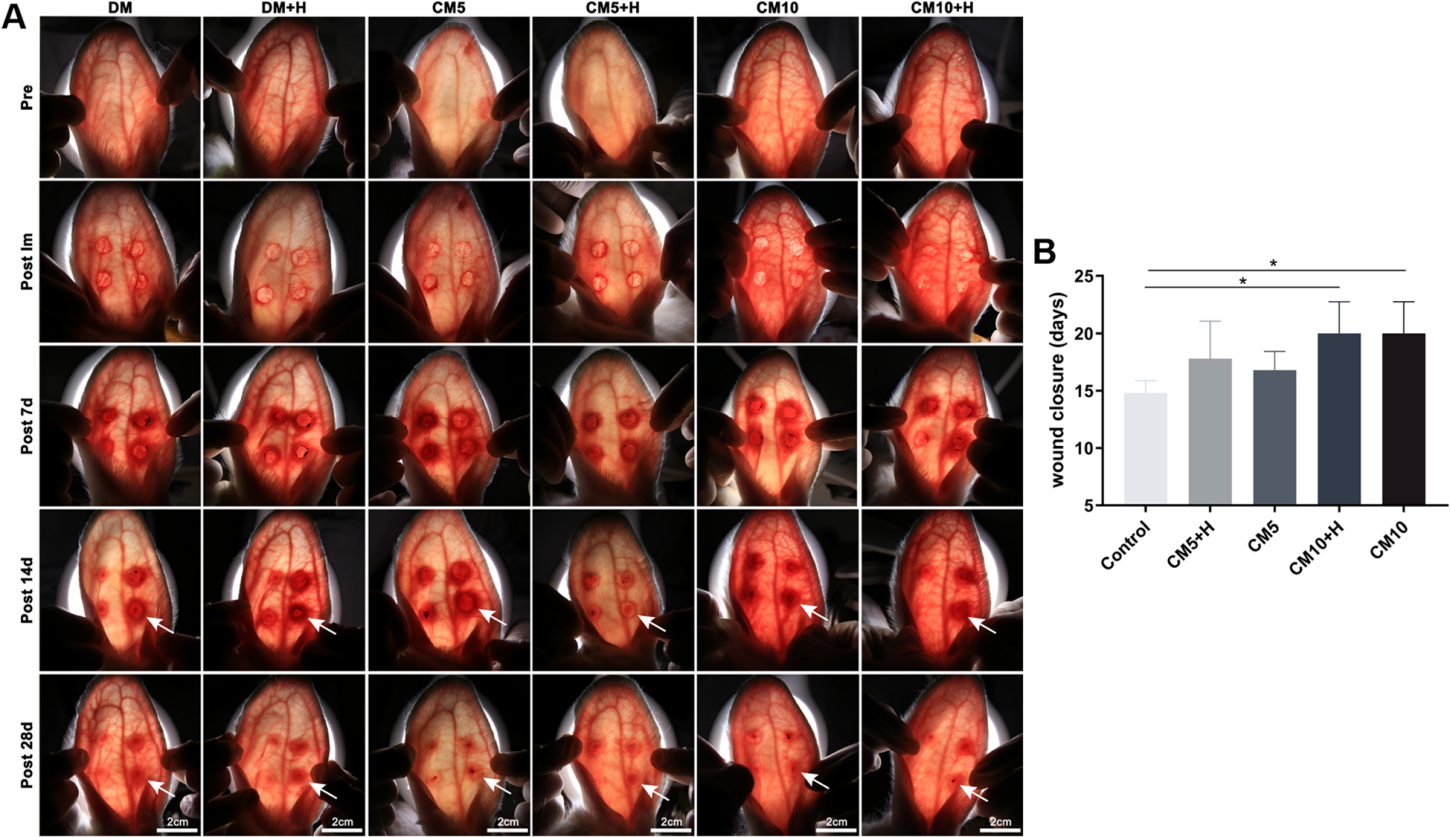
Observation of wound healing. **a** A light source was applied to the rabbit ears’ dorsal side to observe the wound redness and angiogenesis. The area depicted by the arrow refers to the edge of the wound. Pre, preoperative; Post Im, postoperative immediately. **b** Comparison of wound closure time in each group. Blank was set as control

In the follow-up observation, the hyperemia, redness, and swelling of the wound in the CM5+H group became mild on the 14th day. In contrast, in the DMEM, DMEM+H, CM5, CM10, and CM10+H groups, the inflammation and proliferative wound healing phase were prolonged (Fig. 4a). In the process of tissue regeneration, there was a delay in wound closure in the CM10 and CM10+H groups, compared with the blank control group (*p* < 0.05), and no significant variation was found in healing time between the gel group and the non-gel group (Fig. 4b).

### The inhibitory effect of polysaccharide hydrogel combined with ADSCC-CM on scar proliferation

**Fig. 5:**
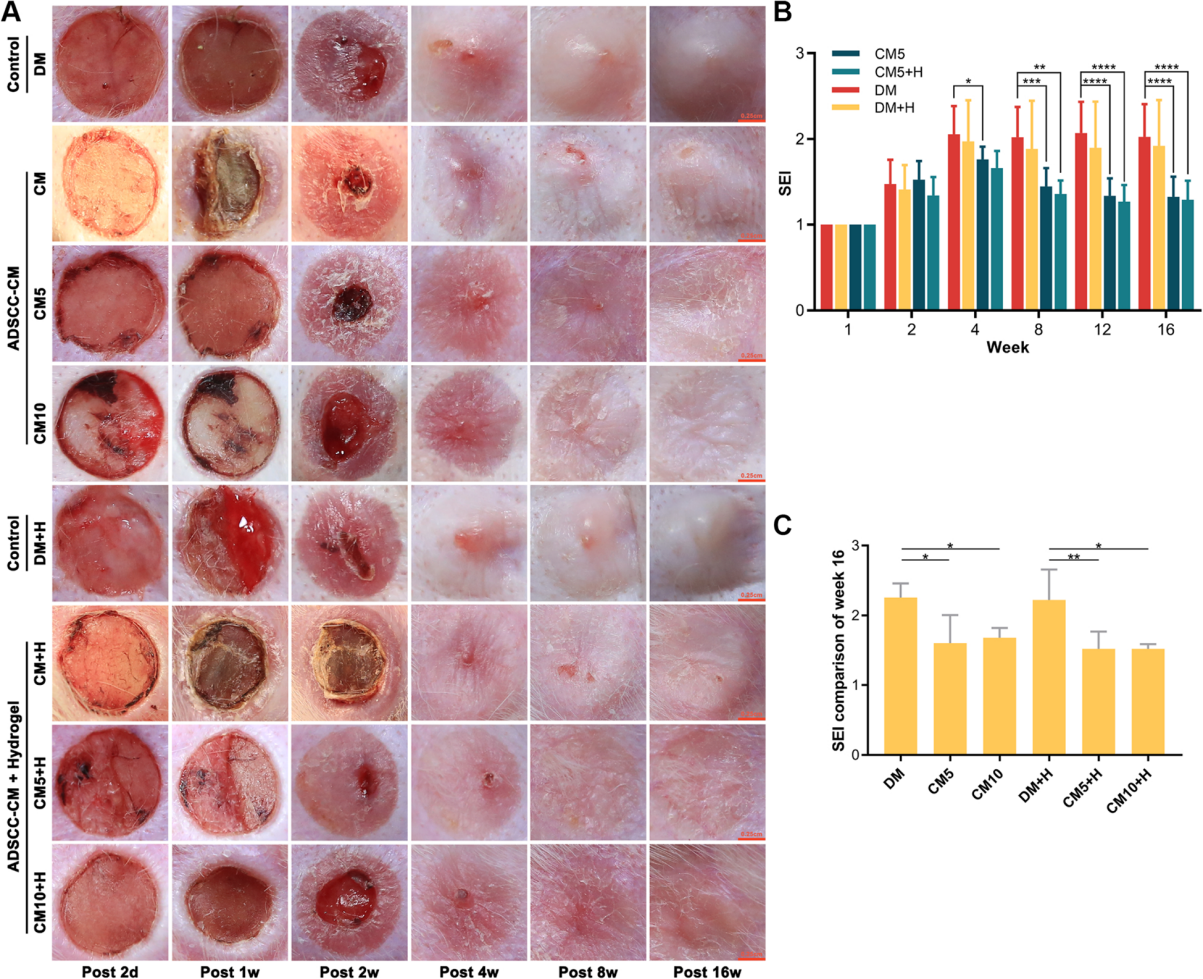
Observation of scar hyperplasia. **a** Gross view of scarring in rabbit ears. **b** Comparison of SEI between different groups (*n* = 8). **c** Comparison of SEI at 16 weeks. The variation was obtained by intra-group comparison between the gel group and non-gel group; *p < 0.05, **p < 0.01, ***p < 0.001, ****p < 0.0001

**Fig. 6:**
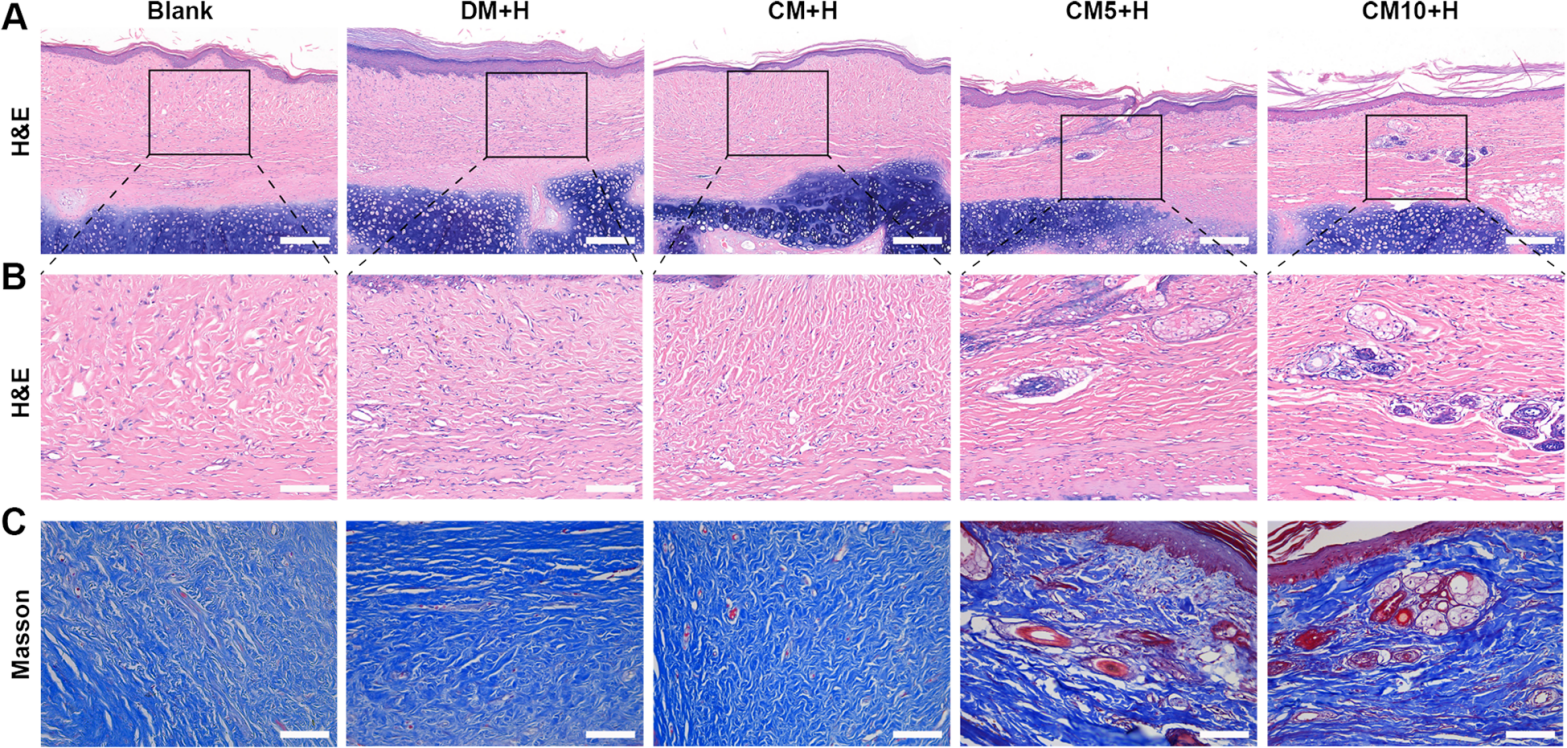
Pathological sections of scar tissue at 16 weeks. **a**, **b** H&E staining was selected to assess the full-thickness skin section. Scale bar 200 μm in original images and 100 μm in magnified images. **c** Masson’s staining revealed the arrangement of collagen fibres. Scale bar 100 μm

We noted a corresponding decrease in the growth of scar in the CM5, CM5+H, CM10, and CM10+H groups with reduced SEI. Among them, CM5+H yielded the best preventive effect on scar hyperplasia, with higher-quality scarring. Hyperemia, redness, and swelling subsided more rapidly at the early stage of tissue regeneration. Melanin, height, vascularity, and pliability were better than those of the control group. (Fig. 5a–c). In the control group, large collagen fibres were deposited, accompanied by a disordered collagen fibre arrangement, with barely any skin appendage regeneration. In comparison, collagen deposition was decreased, with uniform collagen distribution and visible skin appendage regeneration in the CM5+H and CM10+H groups (Fig. 6a–c).

### Proteomic analysis

**Fig. 7:**
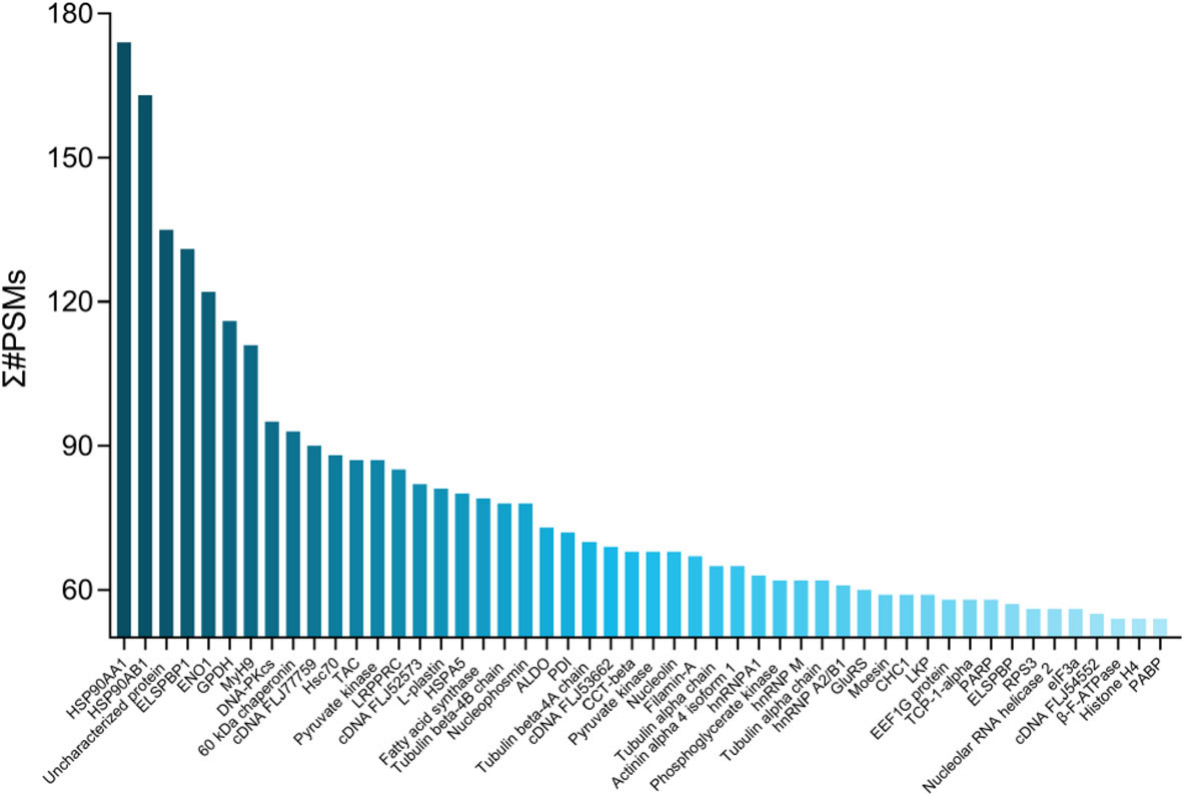
Protein mass spectrometry detection of ADSC-CM. The top 50 proteins with higher relative abundance in adipose stem cell-conditioned medium were obtained in shotgun LC-MS/MS analysis

Shotgun LC-MS/MS analysis was performed for mass protein detection of ADSC-CM. Consequently, 12,221 peptides and 2349 proteins were obtained (Additional file 2: Table S1–2), and the top 50 proteins with high relative abundance (Σ#PSMs) were preliminarily evaluated (Fig. 7). Among them, heat shock protein 90 kDa α (HSP90 α) is a potential factor driving the wound’s expected closure [24], while deficiency of protein disulfide isomerase (PDI), resulting in trauma-related migration and recovery area [25]. Tubulin alpha chain (TAC) may be involved in PLAB-mediated apoptosis of hypertrophic scar fibroblasts, and elongation factor 1-α 1 (EF-1α) potentially participate in scarless healing [26, 27]. Collectively, the co-existence of HSP90 α, PDI, TAC, and EF-1α in the supernatant of stem cells may be positively correlated with tissue repair and scar prophylaxis.

## Discussion

Upcoming clinical practice enrolling ADSCs, stromal vascular fraction cells (SVFs), and platelet-rich plasma (PRP) has started to illuminate a role for exogenous cytokines in wound healing and scarring [28–30]. A variety of cell growth factors such as transforming growth factor-β3 (TGF-β3), interleukin-10 (IL-10), and basic fibroblast factor (bFGF) have been found to promote tissue cell repair and wound healing [31]. With ascending evidence in the field of the validity of stem cell supernatant in remission of scarring [32, 33], there is a good rationale for pursuing the development of ADSCC-CM as new potential therapeutic agents.

Notably, α-SMA and collagen deposition related to the activity of the TGF-β/Smad2 signalling pathway participates in the transition of fibroblasts/myofibroblasts [34]. In light of this potential to affect the pathophysiological processes in HS development, the alleviation of α-SMA expression also illustrates a suppressive role in scarring. As the potential inhibitor of hypertrophic scarring, the maintenance of the therapeutic concentration of cytokines in stem cell-conditioned medium ensures the sustainability of clinical outcomes.

As depicted in the illustrations, we test the hypothesis that ADSCC-CM can alter the α-SMA expression of HKFs and inhibit scar formation in a dose-dependent manner. The CM and CM5 groups could accelerate fibroblasts’ proliferation, but such phenotype would be reversed when the concentration further arose. This interaction may help explain the delay of wound closure in the CM10 and CM10+H groups. While CM has a limited inhibitory effect on α-SMA and could not withhold the growth of scar, reaching the concentration of CM5 and CM10 can help lower the expression of α-SMA in HKFs. Dose-dependently, the CM5 and CM10 groups come into effect of suppressing the forming of a scar.

To date, there has been an exponential surge in the rise of tissue engineering. Bioengineering strategies are inclined to the combination of biological materials, cells, and bioactive factors in conformity with tissue regeneration [35]. With the complement of tissue engineering, stem cell therapy can overcome some of the existing shortcomings. For instance, hyaluronic acid encapsulation reinforced the survival and efficacy of transplanted stem cells in wound healing [36]. The scaffold can not only provide mechanical support but also serve as the niche of mesenchymal stem cells by improving their paracrine activity [37, 38]. As a commonly used biological material in clinical practice, polysaccharide hydrogel interacts with the Ca^2+^ or Na^+^ from the conditioned medium to form a network structure [39]. We can compound any required cytokines or seed cells in the hydrogel delivery system in the liquid state [40]. The injectable hydrogel can be transferred locally or transplanted to the injured site, exerting its therapeutic effects on wound repair [41]. This drug delivery platform could provide insights into the emerging cell-free strategies as a practically non-invasive therapy for the accurate matching of irregularly shaped tissue defects of the wound surface.

In our research, ADSCC-CM combined with hydrogel exert better influence than that of ADSCC-CM alone in the CM5+H and CM10+H groups, which enables the rearrangement of collagen fibres. The union of the ADSCC-CM and hydrogel may render a semi-solid drug reservoir’s rhythmic forming to exert a slow-release effect, enhancing the scar’s quality. As the potential inhibitor of hypertrophic scarring, the maintenance of the therapeutic concentration of cytokines in stem cell-conditioned medium ensures clinical outcomes sustainability.

Mice can regenerate new hair follicles after full-thickness excision of skin wounds, known as wound-induced hair neogenesis (WIHN) [42]. In the current study, we presented the partial regeneration of skin appendages after ADSCC-CM treatment, since SVF, PRP, and ADSCC-CM can all promote wound healing and hair regeneration [43]. It is conceivable that certain commonalities lay the foundations for overall efficiency. A correlation could be developed based on the following assumptions: the signals of growth factors prolong the growth phase (fibroblast growth factor-7), facilitate hair follicle development (β-catenin), and inhibit apoptosis [44]. Much of this may be attributed to the activation of the Wnt signalling pathway [45], exerting considerable effects on hair growth.

Currently, attempts to pinpoint the crucial signal pathways leading to pathological scar formation initially focused on chemokines and cytokines [46]. Using protein mass spectrometry, we also made a preliminary evaluation of the relevant cytokines that may be curative in the supernatant of stem cells. A handful of proteins with a relatively high abundance were detected, such as HSP90AA1, HSP90AB1, MyH9, and ENO1, which was consistent with Nakashima’s research on human ADSC-CM [47]. Among them, Hsp90α and PDI may be involved in wound repair [24, 25], while TAC and EF-1α are possibly related to scar inhibition [26, 27]. Msh homeobox 2 is also a pivotal element, further amplifying the Wnt signal integrant to WIHN [48].

This study’s limitations lie in the uncertainty of the specific factors responsible for skin regeneration in ADSCC-CM. The research on the mechanism of ADSCC-CM induced scar alleviation should be conducted for future reference. Also, no significant variation emerged in the acceleration of re-epithelialization. Whether the freeze-drying method will lead to the inactivation of specific proteins in ADSCC-CM remains to be further explored. To date, a new model of rat tail hypertrophic scar was demonstrated, which is analogous to both normal-trophic and hypertrophic scarring in humans [49]. Alternatively, this model could be utilized for follow-up in-depth research, which might be a solution for the lack of antibodies in rabbit species.

## Conclusions

In summary, lyophilized ADSC-CM contains a variety of proteins related to tissue repair and scar formation. Lyophilized mesenchymal stem cell concentrated conditioned medium implies a pivotally suppressive role for scar proliferation. As a stable drug delivery system, the combination of ADSCC-CM and polysaccharide hydrogel may slow-release functional proteins to suppress scar growth. Among them, CM5+H had the best preventive effect on scar hyperplasia by boosting the growth and survival of fibroblasts, downregulating the expression of fibroblast α-SMA, and promoting the rearrangement of collagen fibres concurrently, which would be a novel approach to scar hyperplasia prophylaxis.

## Supplementary Information

**Additional file 1: Figure S1.** The graphical abstract of ADSCC-CM combined with polysaccharide hydrogel: The concentration of therapeutic cytokines in stem cell-conditioned medium was elevated by lyophilization and rehydration. Simultaneously, the ionic molecules in the conditioned medium helped connect the short nanofibers in the polysaccharide hydrogel, forming a semi-solid drug reservoir for scarring’s remission. **Figure S2.** Freeze-drying of ADSC-CM: (A)Complete lyophilization of ADSC-CM in the tested volume of 10 ml and 15 ml. (B) Comparison of the ADSC-CM weight before and after freeze-drying.

**Additional file 2: Table S1-2.** Shotgun LC-MS/MS analysis of ADSC-CM: The protein solution separated by SDS-PAGE were digested into peptide mixtures, which can be matched in the corresponding database. Through the assembling of the complete series of each protein in the mix, each protein was identified.

## Data Availability

The data that support the findings of this study are available from the corresponding author upon reasonable request.

## Abbreviations

ADSCs: Adipose-derived stem cells
rADSCs: Rabbit adipose-derived stem cells
ADSC-CM: Adipose-derived stem cell conditioned medium
ADSCC-CM: Adipose-derived stem cell concentrated conditioned medium
bFGF: Basic fibroblast factor
CM: Conditioned medium of the adipose-derived stem cells
CM5: Five times concentrated conditioned medium of the adipose-derived stem cells
CM10: Ten times concentrated conditioned medium of the adipose-derived stem cells
CM+H: CM mixed with polysaccharide hydrogel
CM5+H: CM5 mixed with polysaccharide hydrogel
CM10+H: CM10 mixed with polysaccharide hydrogel
DMEM: Dulbecco’s modified Eagle’s medium
DPBS: Dulbecco’s phosphate-buffered saline
DAPI: 4′,6-Diamidino-2-phenylindole
EF-1α: Elongation factor 1-α
FBS: Foetal bovine serum
HSP90 α: Heat shock protein 90 kDa α
HKFs: Human keloid fibroblasts
HS: Hypertrophic scar
IL-10: Interleukin-10
PRP: Platelet-rich plasma
PBS: Phosphate-buffered saline
PI: Propidium iodide
PDI: Protein disulfide isomerase
SVFs: Stromal vascular fraction cells
SEI: Scar elevation index
α-SMA: α-Smooth muscle actin
TAC: Tubulin alpha chain
TGF-β3: Transforming growth factor-β3
WIHN: Wound-induced hair neogenesis
