# Supplementary figures and images for: Combination of lyophilized adipose-derived stem cell concentrated conditioned medium and polysaccharide hydrogel in the inhibition of hypertrophic scarring
Source: Stem Cell Res Ther. 2021 Jan 7;12:23. doi: 10.1186/s13287-020-02061-3 (PMC7792059; doi:10.1186/s13287-020-02061-3)

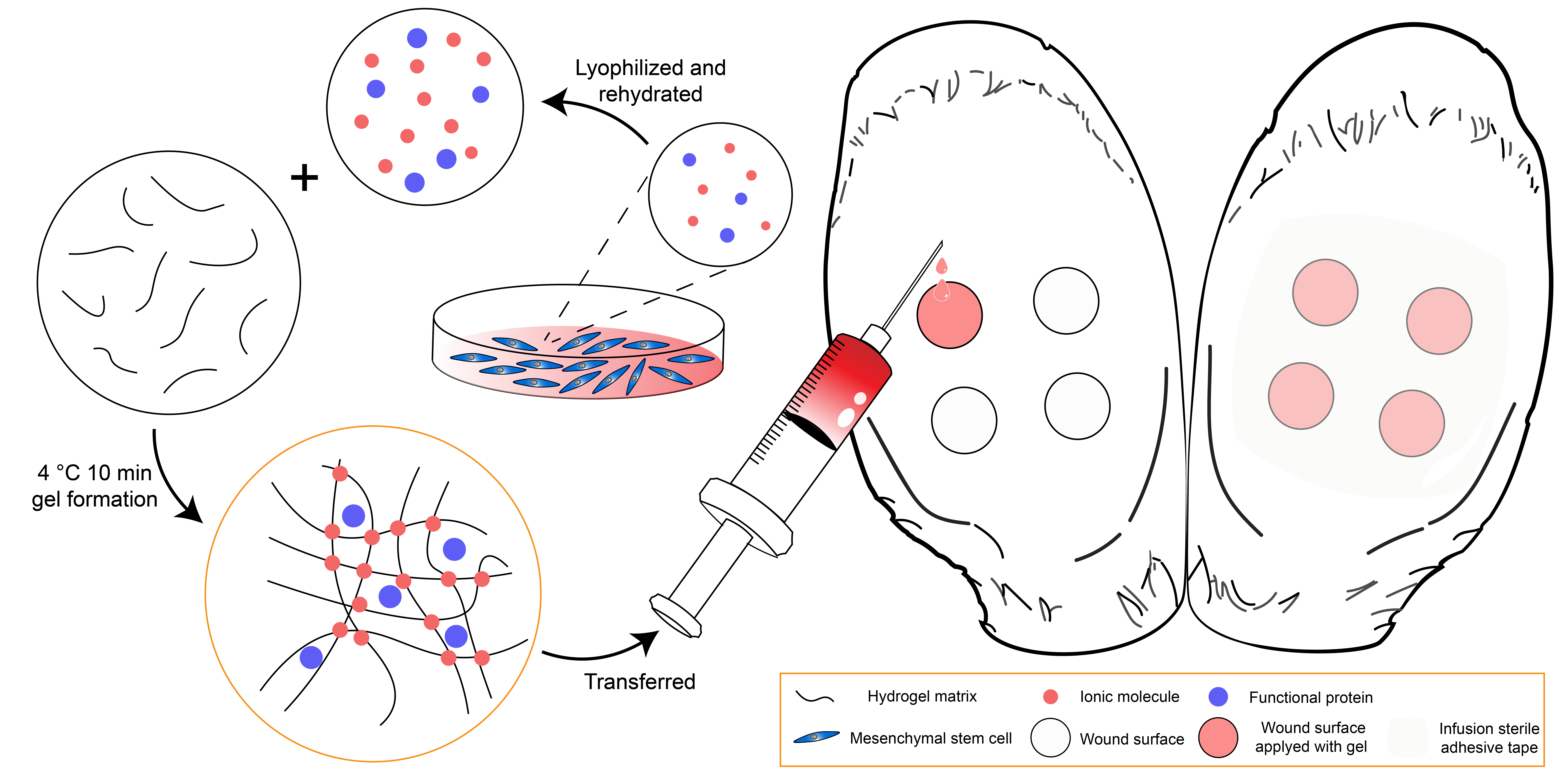

Supplement: Supplementary file 1 — Additional file 1: Figure S1. The graphical abstract of ADSCC-CM combined with polysaccharide hydrogel: The concentration of therapeutic cytokines in stem cell-conditioned medium was elevated by lyophilization and rehydration. Simultaneously, the ionic molecules in the conditioned medium helped connect the short nanofibers in the polysaccharide hydrogel, forming a semi-solid drug reservoir for scarring’s remission. Figure S2. Freeze-drying of ADSC-CM: (A)Complete lyophilization of ADSC-CM in the tested volume of 10 ml and 15 ml. (B) Comparison of the ADSC-CM weight before and after freeze-drying. [file 13287_2020_2061_MOESM1_ESM.zip › Additional file 1 Figure S1.png]

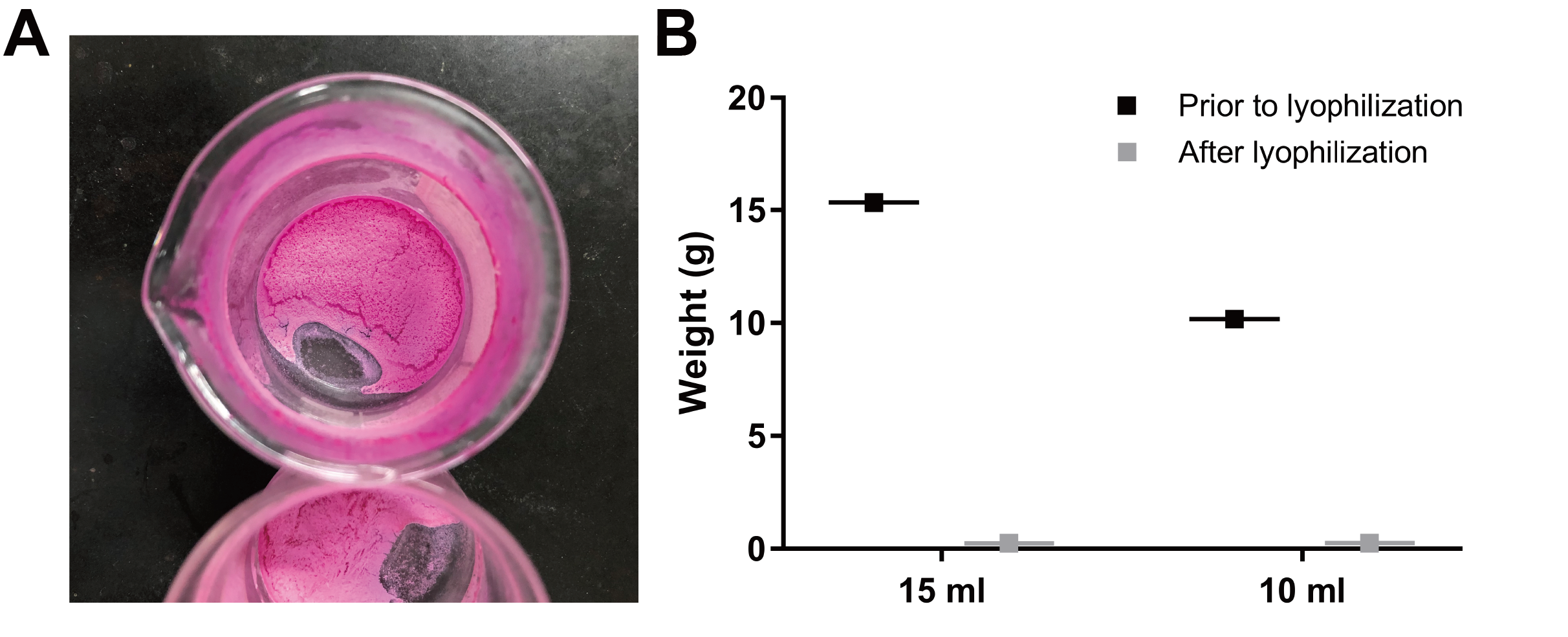

Supplement: Supplementary file 1 — Additional file 1: Figure S1. The graphical abstract of ADSCC-CM combined with polysaccharide hydrogel: The concentration of therapeutic cytokines in stem cell-conditioned medium was elevated by lyophilization and rehydration. Simultaneously, the ionic molecules in the conditioned medium helped connect the short nanofibers in the polysaccharide hydrogel, forming a semi-solid drug reservoir for scarring’s remission. Figure S2. Freeze-drying of ADSC-CM: (A)Complete lyophilization of ADSC-CM in the tested volume of 10 ml and 15 ml. (B) Comparison of the ADSC-CM weight before and after freeze-drying. [file 13287_2020_2061_MOESM1_ESM.zip › Additional file 1 Figure S2.png]
